# Decrypting cryptic pockets with physics-based simulations and artificial intelligence

**DOI:** 10.1016/j.sbi.2025.103215

**Published:** 2026-01-27

**Authors:** Si Zhang, Gregory R. Bowman

**Affiliations:** Department of Biochemistry and Biophysics, University of Pennsylvania, Philadelphia, PA 19104, USA

## Abstract

Cryptic pockets are promising targets for drug discovery that greatly expand the druggable proteome. In particular, they can provide opportunities to target proteins previously thought to be “undruggable” due to a lack of pockets in structures of the ground state. However, their transient and hidden nature renders them difficult to detect through conventional experimental screening methods. Recent advances in computational methodologies and resources have greatly enhanced our ability to identify and characterize such elusive pockets. This review highlights key developments in computational approaches, including physics-based molecular dynamics simulations, artificial intelligence–driven models, and hybrid strategies that integrate both to enhance cryptic pocket discovery and functional interpretation.

## Introduction

Cryptic pockets are transient binding sites that arise from conformational rearrangements driven by a protein’s thermal fluctuations. Opening of these pockets is rare, so they are typically closed, and therefore hidden, in experimental structures. This situation is likely exacerbated by the fact that most protein structures are determined under cryogenic conditions (~100 K), which severely restrict conformational flexibility [1,2]. As a result, identifying cryptic pockets is challenging even though they are thought to be quite common [3,4]. Most known cryptic sites were discovered serendipitously by solving structures for hits from high-throughput screens that happened to find molecules that bind and stabilize an open cryptic pocket [5–8]. It would be far better to have a means to discover cryptic pockets without needing molecules that bind them. Then one could use this knowledge of a pocket’s structure/location to intentionally target the pocket.

The ability to identify and target cryptic pockets would provide a host of new opportunities for drug discovery. In particular, they could provide a means to target proteins historically deemed “undruggable” due to lack of apparent binding pockets. Targeting cryptic pockets could provide greater specificity than targeting functional sites (e.g. for kinases, where molecules that target the active site of one kinase are likely to inhibit other kinases). Many cryptic pockets are allosterically coupled to functional sites, opening up the possibility of enhancing desirable functions in addition to inhibiting undesirable functions [8–14].

Despite the challenges that cryptic pockets pose, recent successes highlight their therapeutic potential. For example, the discovery of a hidden switch-II pocket in KRAS G12C led to the development of two Food and Drug Administration—approved covalent inhibitors, AMG 510 and MRTX849 [15,16]. These compounds selectively bind to cysteine 12 within a cryptic site. Similarly, in CB1, the discovery of an extended cryptic pocket bridging the orthosteric site and the conserved signaling residue D^2.50^ enabled the rational design of a ligand, VIP36 [17].

**Figure 1:**
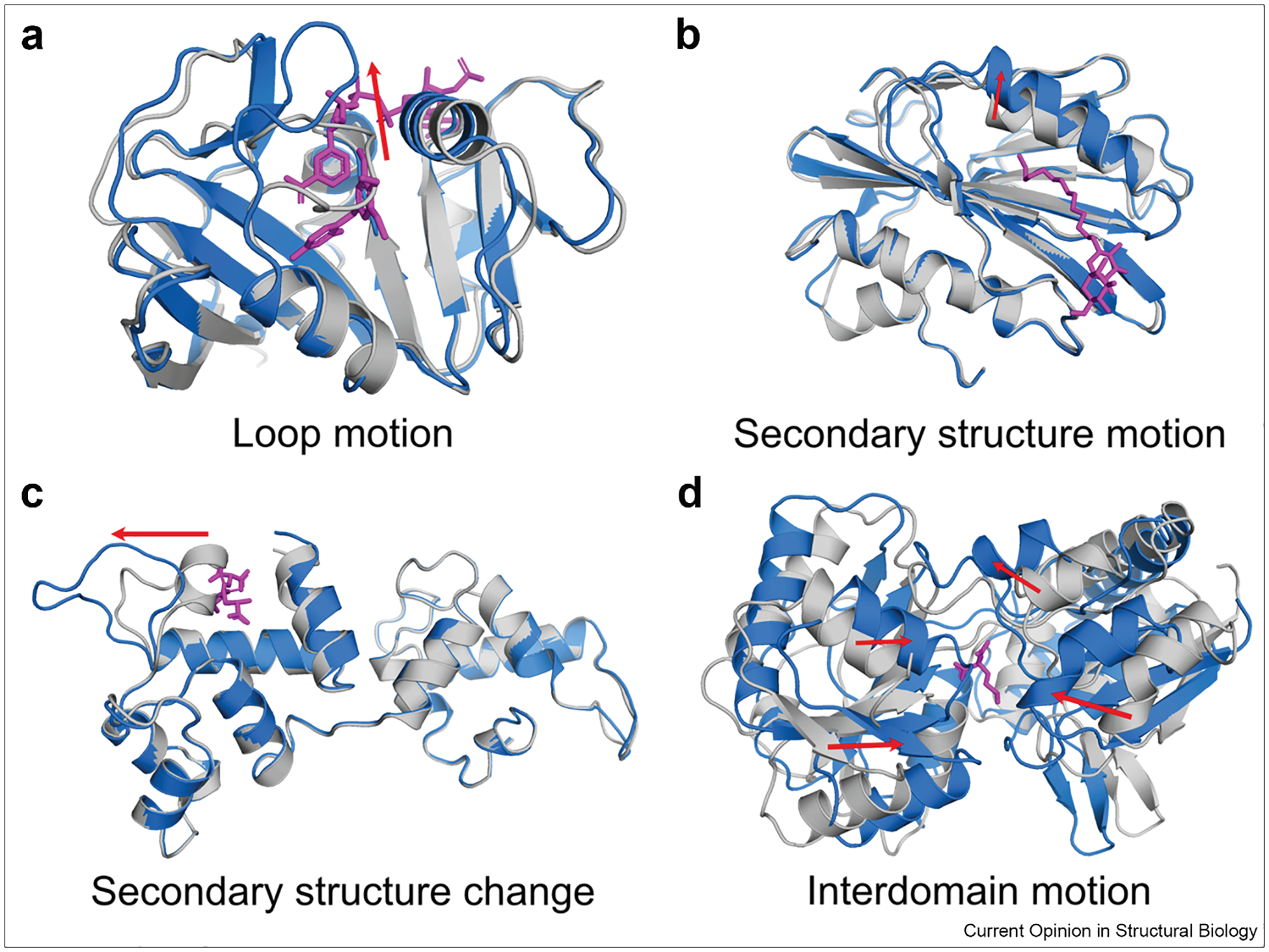
Four representative structural rearrangements leading to cryptic pocket formation. (Adapted from Ref. [3]). Apo structures are shown in gray, holo structures in blue, and ligands in magenta. Red arrows indicate the primary conformational changes between apo and holo states. Cryptic pockets can arise through distinct local or global motions, including A) loop motion as seen in dihydrofolate reductase (apo PDB: 2W9T, holo PDB: 2W9S), B) secondary structure motion exemplified by lipoprotein LpqN (apo PDB: 6E5D, holo PDB: 6E5F), C) secondary structure change, as in calcium- and integrin-binding protein 1 (apo PDB: 1Y1A chain A, holo PDB: 1Y1A chain B), and D) interdomain motion as observed in nopaline-binding periplasmic protein (apo PDB: 4POI, holo PDB: 5OTA). PDB, Protein Data Bank.

Advances in computational methods are helping to make the discovery and targeting of cryptic pockets more routine. In particular, molecular dynamics (MD) simulations offer a powerful means to capture protein dynamics and reveal transient structural changes that create cryptic pockets [17,18]. Such motions, ranging from side chain and loop fluctuations to interdomain rearrangements, occur across diverse timescales as illustrated in Figure 1. Enhanced sampling techniques can further improve detection of rare, high-energy conformational states that are critical for the emergence of cryptic sites [19–21]. Meanwhile, advances in artificial intelligence (AI), such as AlphaFold (AlphaFold3) [22,23], have added new capabilities to structure prediction and cryptic pocket exploration [24,25].

Hybrid approaches combining physics-based simulations with machine learning models, such as PocketMiner [3] and BioEmu [26], have opened new avenues for structural and functional predictions.

**Figure 2:**
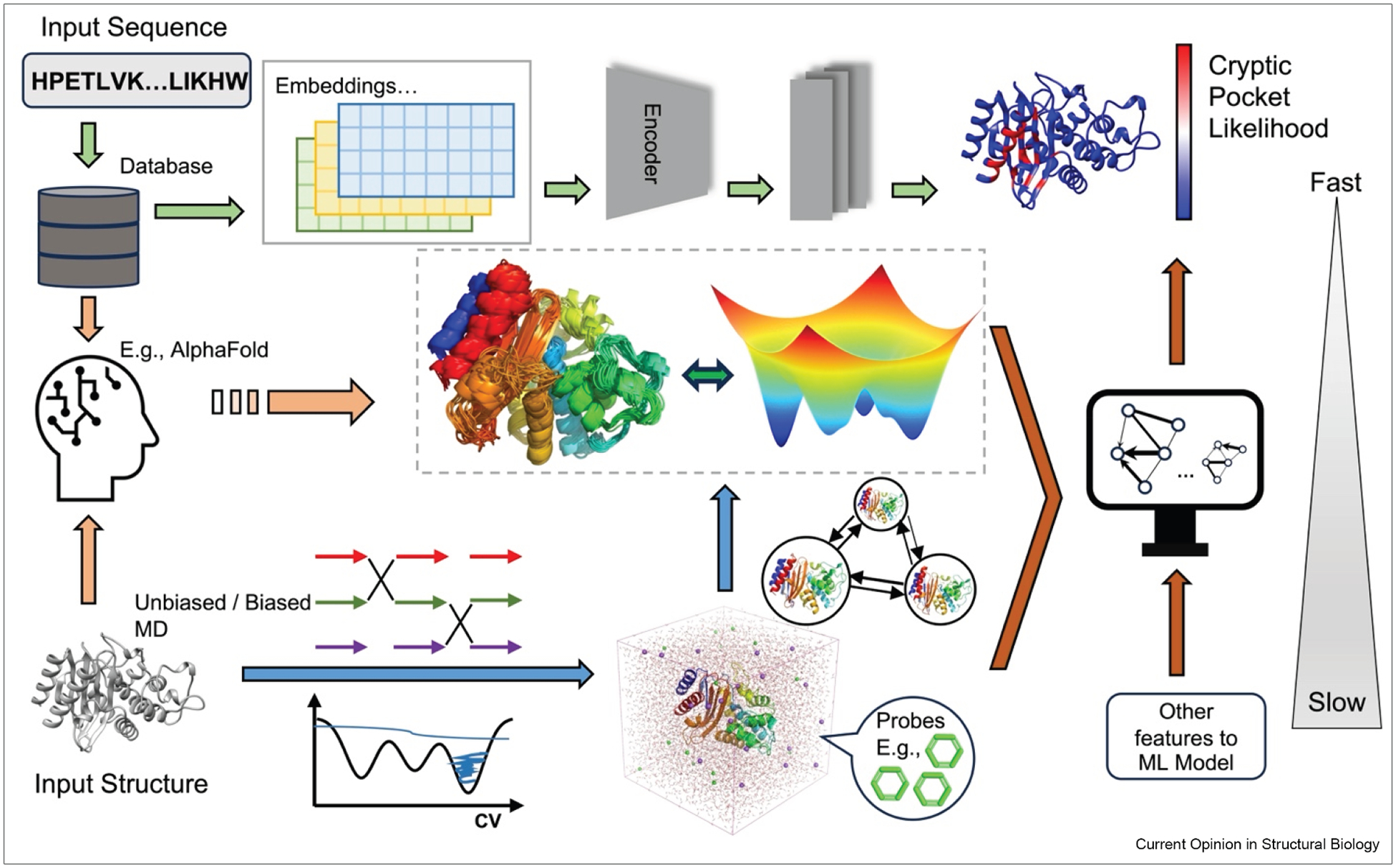
Overview of computational approaches to investigate cryptic pockets. Cryptic pockets can be explored using MD simulations or enhanced sampling techniques to generate structural ensembles with detailed conformational information. MSMs are often applied at this stage to analyze conformational landscape. Alternatively, cryptic pockets can be predicted using AI-driven models, such as AlphaFold or sequence-based neural networks. By combining simulation data with machine learning, residue-level predictions of pocket-forming regions are also possible. MD, molecular dynamics; MSMs, Markov state models.

Here, we review recent progress in uncovering cryptic pockets (Table 1, Figure 2) using molecular simulations and AI-driven models (individually and in combination) and explore how these tools are expanding our ability to identify cryptic site formation and their implications for drug discovery.

## Exploring cryptic pockets through molecular simulations

MD simulations have become a powerful technique for investigating cryptic pockets in proteins [17,18,27–30]. In early studies, docking was directly performed on MD-generated snapshots containing transient pockets to assess ligand binding potential and pocket properties. However, even long-timescale MD simulation, spanning hundreds of microseconds, can fall short in capturing transient, high-energy pocket states in certain systems.

For instance, pockets that emerge from secondary structure motions or changes may occur on timescales ranging from microseconds to minutes, often exceeding the practical limits of conventional MD simulations.

To overcome these limitations and accelerate the exploration of relevant conformational space, enhanced sampling techniques, such as the fluctuation amplification of specific traits (FAST), have been introduced. FAST efficiently guides simulations toward conformations exhibiting desired structural features, like increased inter-residue distances or increased pocket volumes, by iteratively running simulations, building a map of the space explored so far, and using that map to decide where to gather more data [46].

Markov state models (MSMs) are commonly used to map the conformational landscape sampled by MD [31–36]. MSMs discretize this landscape into a finite set of states, from which representative conformations with well-defined cryptic pockets can be extracted. Ligand binding can then be evaluated using advanced methods such as Boltzmann docking [47] and PopShift [48], both of which account for the probabilities of different states and how strongly a ligand binds each state. This MSM-based framework has been proven to be effective for modeling the binding of known ligands that target alternative sites (e.g., orthosteric regions) and for virtually screening compound libraries against cryptic pockets. Notably, it has enabled the successful discovery of ligands for targets like TEM-1 β-lactamase and the 5-HT_3A_ receptor [14,36].

**Figure 3:**
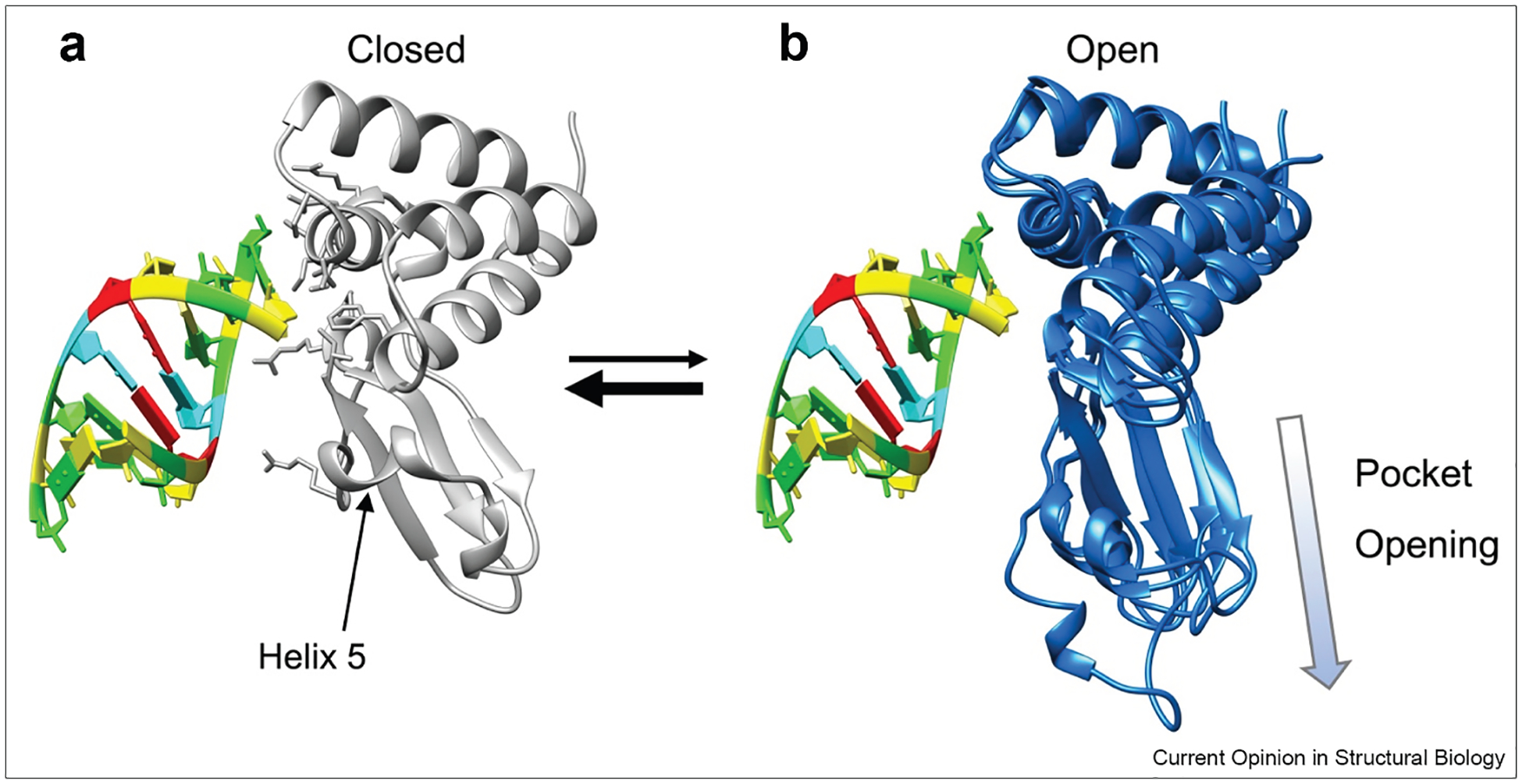
VP35 open and closed conformations. A) Crystal structure of VP35 (gray, chain B) bound to dsRNA (colored using the standard Nucleic Acid Database scheme) via the flat interface (PDB: 3L35), representing the closed conformation. VP35 residues in contact with dsRNA are shown as sticks. B) Three representative MD-simulated structures showing the opening of the cryptic pocket via the displacement of helix 5. These structures are superimposed onto the crystal VP35 (not shown) and the dsRNA to highlight the conformational changes. PDB, Protein Data Bank.

A particularly compelling example of this framework is the discovery of a cryptic pocket in the interferon inhibitory domain of Zaire Ebola VP35 (VP35) [34]. A combination of FAST and large-scale simulations on the Folding@home distributed computing platform [49,50] were used to explore the protein’s conformational space. To identify cryptic pocket within this large ensemble, exposon analysis was employed [51]. Exposons are groups of residues that undergo highly correlated changes in solvent exposure and are often associated with cryptic site formation. This analysis revealed a cryptic pocket that forms when a small helix (helix 5) separates from a 4-helix bundle (Figure 3). This pocket was found to be allosterically coupled to the double-stranded RNA (dsRNA) blunt-end-binding interface, which was captured in a separate exposon. The correlation of all rotameric and dynamical states algorithm [52] further validated this allosteric connection by quantifying the coupling between residue pairs through their dihedral angle dynamics. To gain mechanistic insight, the DiffNets machine learning algorithm [53], a supervised autoencoder architecture designed to identify key structural differences between ensembles (e.g. open vs. closed states), was applied. DiffNets revealed strong coupling between cryptic pocket opening/closing and the structural preferences of a key RNA-binding residue, F239. Subsequent experiments confirmed the existence of the pocket and its allosteric control over RNA binding. This study represents a rare example of successfully targeting a difficult, nonenzymatic protein involved in protein—nucleic acid interactions, demonstrating the therapeutic potential of leveraging cryptic pockets to modulate such challenging targets. A follow-up study demonstrated that the open state of the pocket has a biological function, which is an exciting insight [54].

Metadynamics is another promising approach for studying cryptic pockets. It is particularly effective at enhancing sampling along predefined conformational coordinates, known as collective variables (CVs), thereby facilitating access to otherwise rare structural states [41]. For example, Benabderrahmane et al. systematically identified cryptic pockets in the antiapoptotic protein, Mcl-1 by performing well-tempered metadynamics on essential coordinate space [42]. These coordinates, used as CVs, were derived from essential dynamics captured via principal component analysis of an initial 100-ns unbiased MD simulation, allowing enhanced sampling simulations to focus on the dominant protein motions.

More recently, Vithani et al. applied an advanced variant of weighted ensemble (WE) MD simulations to investigate cryptic pockets in both wild-type KRAS and its G12D mutant [19]. In their approach, normal mode analysis was used to derive inherent normal modes, which then served as progress coordinates to guide simulations toward relevant conformational transitions. Notably, their study also incorporated mixed-solvent MD (MSMD) simulations, in which small cosolvent molecules were introduced as probes to identify hydrophobic or transiently accessible cavities. MSMD not only enables the detection of cryptic sites but also provides quantitative characterization of pocket properties, such as estimated binding free energies through cosolvent occupancy analysis [37–40,55]. Its growing popularity stems from its ability to mimic ligand—induced-fit effects and uncover dynamic binding hotspots that may take longer to open in aqueous-only simulations. Despite its strengths, MSMD alone has limitations, including challenges in achieving sufficient sampling, risks of protein destabilization, and the need for careful selection of probe molecules [56]. To overcome these issues and harness the strengths of MSMD, it is increasingly being integrated with other simulation techniques. In this study, Vithani et al. combined MSMD with WE simulations using xenon as the chemical probe [19], allowing simultaneous capture of induced-fit dynamics and quantification of cosolvent occupancy and residence times. Leveraging over 400 μs of simulation data, the authors conducted comprehensive analyses of cryptic pockets in both KRAS and its G12D mutant. The analyses included probe occupancy mapping, exposon analysis, and a modified version of exposon termed as dynamic probe binding analysis, which calculates the correlated changes in xenon binding. Together, these approaches provided mechanistic insights into pocket flexibility and revealed key allosteric networks with respect to the cryptic pockets in KRAS.

Another promising method is Sampling Water Interfaces through Scaled Hamiltonians (SWISH), a Hamiltonian Replica Exchange (HREX)-based technique devised by Oleinikovas et al. [21]. In combination with small organic probes, SWISH progressively scales the nonbonded interactions between solvent molecules and apolar protein atoms, effectively shifting the water properties toward more ligand-like behavior and thereby facilitating the opening of cryptic sites. This approach has been shown to explore conformational changes with high activation barriers and successfully induce the formation of known cryptic binding sites in targets TEM-1 β-lactamase, interleukin-2, and Polo-like kinase-1. Compared to conventional long-timescale MD or parallel tempering simulations, SWISH offers improved accuracy and sampling efficiency for uncovering cryptic conformations. Building on this, the same group recently developed SWISH-X, an enhanced version of SWISH that combines OPES MultiThermal for faster and more accurate cryptic pocket exploration across diverse systems [20].

While these approaches show strong potential for cryptic pocket detection, their application in drug discovery to unexplored proteins still requires extensive downstream analysis. This includes quantitative characterization of pocket properties, such as assessing pocket druggability and functional relevance [9,57–62], as well as experimental tests using methods like thiol labeling or fragment-based screening [32,34,63,64].

## Identifying cryptic pockets using AI-driven models

With the rapid advancement of AI, a new generation of AI-driven models has emerged to tackle the challenges of cryptic pocket detection. These models can either predict the likelihood of individual residues participating in cryptic pockets or generate open pocket conformations far more efficiently than conventional MD simulations.

Structure prediction tools like AlphaFold have demonstrated remarkable accuracy in modeling protein structures [22]. Through stochastic sampling of input multiple sequence alignments, AlphaFold can produce structural ensembles that may exhibit open or partially open cryptic pockets. However, despite its success, AlphaFold was primarily trained on experimentally determined structures from the Protein Data Bank (PDB) and large sequence databases. It was not explicitly designed to sample alternative conformations, which limits its ability to directly predict cryptic pockets. For instance, a study by Meller et al. showed that AlphaFold could recapitulate cryptic pockets in only 6 out of 10 proteins [24]. The predicted structural ensembles also lack information about the relative weights of different conformations, making it unclear how likely a pocket-opening event is. Additionally, some AlphaFold-predicted structures contain highly flexible domains with low prediction confidence, further limiting applicability. Nevertheless, these predicted structures can serve as valuable starting points for further exploration using unbiased or biased MD simulations [24,25]. In Meller et al.’s study, subsequent MD simulations followed by MSMs analysis provided deeper insights into pocket dynamics that were not accessible from AlphaFold alone. Starting simulations from these predicted structures also accelerated pocket discovery compared to starting from crystal structures. With the recent release of AlphaFold3, which supports joint structure prediction of proteins and small molecules, there is potential for improved identification of open pocket conformations and ligand-induced structural changes [23].

In parallel, sequence-based AI models have recently emerged as powerful tools for protein structure and function prediction, including the assessment of cryptic pocket propensity at the residue level without requiring structural input. Škrhák et al. utilized three different protein language models, ProtT5-XL-U50, ESM-1b, and ProtBert-BFD, to generate residue-level embeddings, which were then fed into a neural network for prediction [43]. These embeddings capture rich contextual information from protein sequences, enabling accurate cryptic site predictions directly from sequence data. Model performance was evaluated using the CryptoSite dataset, which comprises 93 apo—holo protein structure pairs containing validated cryptic pockets [4]. Notably, predictions based on ProtT5-XL-U50 and ESM-1b embeddings outperformed ProtBert-BFD and slightly surpassed CryptoSite, a structure-based model, in terms of the area under the curve (AUC) on the test set. To support the development and evaluation of cryptic pocket predictors, the same group introduced CryptoBench, a larger and more comprehensive benchmark set consisting of 1107 apo—holo protein pairs curated using pocket RMSD (root mean square deviation) as the selection criterion [44]. Using this dataset, they trained a new neural network model using embeddings from the ESM2–3B model. Although this model achieved higher AUC scores on the test set compared to PocketMiner, a structure-based predictor, it is important to note that the two models were trained on different datasets.

Advancing the field further, Martinez et al. constructed the largest known database of cryptic sites to date, comprising over 5.5 million structural alignments of apo and holo protein pairs from the PDB [45]. Using a curated dataset of 71 cryptic and 128 non-cryptic examples, they trained a supervised machine learning model to detect ligand-induced conformational changes and score cryptic pocket formation, ultimately identifying approximately 2,00,000 apo—holo combinations containing potential cryptic sites. Building on this dataset, the authors then fine-tuned a protein language model (Prot-T5-XL-UniRef50) to predict cryptic pocket locations directly from the sequence. Although this model demonstrated high prediction performance when query sequences share over 20 % sequence identity with CryptoBank entries, its generalizability to novel sequences remained limited. Additionally, analyses of ligand molecular weight and relative solvent accessible surface area (RSA) revealed that many cryptic pockets tend to accommodate larger ligands (molecular weight >300 Da) and are located in deeper, less solvent-exposed regions (RSA <0.3) rather than on the surface (RSA >0.3). Based on these insights, they curated a refined fragment library of ~ 6000 clustered ligands designed specifically to screen for cryptic binding pockets.

Together, these AI-driven approaches complement physics-based simulations by enabling rapid screening and prioritization of potential cryptic sites, especially valuable for targets with limited structural information or where experimental data are sparse.

## Integrating AI with simulation data to predict cryptic pockets

In addition to sequence-based models, structure-based machine learning models represent another powerful class of tools for cryptic pocket predictions, leveraging protein structural ensembles sampled from MD simulations. A notable early example is CryptoSite, which requires an input protein structure and was trained to identify residues that transition from an orientation incompatible with ligand binding to one that accommodates a ligand [4]. Its training was based on a set of 84 confirmed cryptic pockets derived from the PDB. While CryptoSite achieves good accuracy in classifying pocket-forming residues, its application is computationally expensive―it requires generating simulation data on-the-fly as one of the input features, taking approximately one day per input structure.

A more recent and efficient alternative is PocketMiner, which predicts whether each residue in a given protein structure will participate in the formation of a cryptic pocket during a short MD simulation initiated from that structure [3]. The prediction is typically generated within seconds. PocketMiner is built on a geometric vector perceptron (GVP)-based graph neural network, designed to learn residue-level representations from diverse protein conformations sampled through simulations. For each input structure, the model extracts structural features, such as dihedral angles and inter-residue directions/distances, processes them through GVP layers, and updates the residue embeddings via message passing layers. The final residue-level predictions are obtained using a sigmoid activation function. The model was trained on a curated dataset of 38 proteins containing 39 experimentally confirmed cryptic pockets. A large number of simulations were performed on these proteins, capturing thousands of cryptic pocket-opening events. From these simulations, residue-level training labels were generated by measuring changes in LIGSITE pocket volume and the maximum fpocket druggability score in the vicinity of each residue. The final dataset provided sufficient structural diversity and reliable dynamic labels to support effective model training. Compared to CryptoSite, PocketMiner achieves slightly higher accuracy, as measured by the area under the receiver operating characteristic curve (ROC-AUC: 0.87 vs. 0.85), while offering over 1000-fold faster prediction speed.

One other promising model is BioEmu, which integrates over 200 *ms* of simulation data, static structures, and experimental protein stabilities through a unique training paradigm [26]. Notably, a significant portion of its training data comes from the large-scale distributed computing resource Folding@home [33]. BioEmu predicts diverse functional motions, including cryptic pocket formation, by generating structure ensembles. In a benchmark of 34 experimentally validated cryptic pocket cases, the model successfully recovered 86 % of holo structures. However, its performance on apo conformations was lower, with only 56 % accurately predicted, highlighting the need for further improvements, especially in modeling unbound structures or better balancing apo and holo representations during training.

## Conclusions

Cryptic pockets present exciting opportunities for drug discovery, for example, they can enable the targeting of proteins that were previously considered “undruggable”. However, their transient and dynamic nature makes them inherently difficult to detect using static structure-based experimental screening. Physics-based MD simulations, particularly when combining with advanced techniques such as FAST, MSMs, mixed-solvent approaches, and enhanced sampling have proven effective in capturing the dynamic formation and properties of these hidden pockets. Meanwhile, AI-driven models offer rapid and scalable alternatives capable of predicting cryptic pocket locations and dynamics with growing accuracy and interpretability. Despite these advances, key challenges remain: MD simulations are computationally expensive and often system-specific, while AI models may yield unphysical results or struggle to generalize across diverse proteins. A major limitation is the lack of large-scale, experimentally tested datasets for benchmarking. Future efforts should focus on building high-quality training data, integrating AI with physics-based methods, improving model interpretability, and continuous pairing predictions with experimental validation to accelerate cryptic pocket discovery and drug design.

## Figures and Tables

**Table 1:** Summary of physics-based simulation methods, AI models, and their combinations used to study cryptic pockets. This table outlines representative approaches leveraging molecular dynamics (MD) simulations, artificial intelligence (AI), or hybrid methods to investigate cryptic pockets across various protein targets.

By molecular dynamics (MD) simulations	Targets	Reference
Identified transient trench/pocket via ligand docking to MD snapshots. Analyzed pocket (volume) using PASS (putative active sites with spheres). Applied ensemble docking. Distinguished native binding poses using FEP (free energy perturbation). Observed pocket-opening conformations.	HIV-1 integrase (IN) P38 MAP kinase BCL-X_L_, IL-2, MDM2 Ras IL-2 Cannabinoid receptor type 1 (CB1)	Schames et al., 2004 [27] Frembgen-Kesner et al., 2006 [28] Eyrisch et al., 2007 [29] Grant et al., 2011 [30] Shan et al., 2022 [18] Rangari et al., 2025 [17]
**Used Markov state models (MSMs) and/or a goal-oriented adaptive sampling algorithm** Identified pocket via LIGSITE and analyzed allostery using mutual information (MI). Detected cryptic pocket and functional motions via Exposon analysis. Mapped allosteric network between pocket and dsRNA-binding interface via CARDS. Employed Adaptive Bandit MD simulations. Detected cryptic pocket in ion channels.	TEM-1 β-lactamase, IL-2, RNase H TEM-1 β-lactamase SARS-CoV-2 proteins Ebola VP35 MetAP-II 5-HT_3A_ receptor	Bowman et al., 2012 [31] Bowman et al., 2014 [32] Zimmerman et al., 2021 [33] Cruz et al., 2022 [34] Moin ST. et al., 2024 [35] Haloi et al., 2025 [36]
**Mixed-Solvent MD simulations** Identified cryptic sites with organic probes, compared to SiteMap. Ranked the sites based on free energy computed from probe occupancy. Identified cryptic pockets utilizing the topological data analysis.	ExoI, NPC2 (Niemann-Pick C2 protein), Staphylococcal nuclease, T4moD, TetR-like transcription factor LfrR, Eg5 kinesin, Cdc4, P38 α SARS-CoV-2 spike glycoprotein Integrins TIE-2, exodeoxyribonuclease I, β-lactamase, NPC2, androgen receptor, dihydrofolate reductase type 1 from TN4003, ferulic acid decarboxylase, fascin, antimethotrexate CDR1–4 graft VHH	Kimura et al., 2017 [37] Zuzic et al., 2022 [38] Ilie wt al., 2023 [39] Koseki et al., 2025 [40]
**CV-dependent enhanced sampling** Metadynamics Run simulations with a range of CVs. Derived CV from PCA analysis. Weighted Ensembles Estimated progress coordinates via NMA. Simulations run with probes.	fibroblast growth factor receptor (FGFR1) Mcl-1 Kras (& G12D)	Herbert et al., 2013 [41] Benabderrahmane et al., 2021 [42] Vithani et al., 2024 [19]
**CV-independent enhanced sampling** SWISH Studied induced-fit effects via combining with probe molecules. SWISH-X Combined with the rapid temperature range exploration of OPES MutliThermal.	TEM-1 β-lactamase, IL-2, PLK1 TEM-1 β-lactamase, Bcl-X_L_, IL-2, MDM2, HPV-11 E2	Oleinikovas et al., 2016 [21] Borsatto et al., 2024 [20]
**By AI models** AlphaFold (2 & 3) Generates an ensemble of target structures that may exhibit open cryptic pockets. Sequence-based models Used protein language model embeddings. Constructed CryptoBench dataset. Constructed CryptoBank dataset.	**Training set** PDB structures and sequence database 93 apo-holo protein pairs (CryptoSite dataset) 1107 apo-holo protein pairs 5.5 million apo-holo-ligand combinations	Jumper et al., 2021 [22] Abramson et al., 2024 [23] Škrhák et al., 2023 [43] Škrhák et al., 2025 [44] Martinez et al., 2025 [45]
**By integrating simulation with Machine Learning models**		
CryptoSites Requires on-the-fly simulations for each input protein. PocketMiner/AE-PocketMiner Makes predictions for a given protein within seconds. BioEmu Predicts diverse conformational states.	93 apo–holo protein pairs 3290 MD-simulated protein structures (from 38 proteins) Over 200 *ms* MD simulation data, static structures, and experimental protein stabilities.	Cimermancic et al., 2016 [4] Meller et al., 2023 [3] Lewis et al., 2025 [26]

CARDS, correlation of all rotameric and dynamical states; CV, collective variable; NMA, normal mode analysis; PCA, principal component analysis; SWISH, Sampling Water Interfaces through Scaled Hamiltonians.
